# Hepatitis E Prevalence among Acute Febrile Jaundice Patients in Senegal, 2008–2020

**DOI:** 10.4269/ajtmh.25-0030

**Published:** 2025-10-09

**Authors:** Elisabeth Thérèse Faye, Arfang Diamanka, Sophie Kang, Aboubacry Gaye, Hyeonseon Ahn, Safietou Sankhe, Bacary Djilocasse Sadio, Ousseynou Sene, Mignane Ndiaye, Moufid Mhamadi, Martin Faye, Oumar Faye, Ndongo Dia, Cheikh Loucoubar, Boly Diop, Mamadou Ndiaye, Ellen Higginson, Cheikh Talla, Ousmane Faye, Amadou Alpha Sall, Moussa Moïse Diagne, Florian Marks, Gamou Fall

**Affiliations:** ^1^ WHO Collaborating Center for Arbovirus and Viral Hemorrhagic Fevers, Virology Pole, Institut Pasteur de Dakar, Dakar, Senegal;; ^2^ Department of Animal Biology, Faculty of Science and Technology, University Cheikh Anta Diop de Dakar, Dakar, Senegal;; ^3^ Epidemiology, Public Health and Impact, International Vaccine Institute, Seoul, South Korea;; ^4^ Epidemiology, Clinical Research and Data Science Department, Institut Pasteur de Dakar, Dakar, Senegal;; ^5^ Epidemiological Surveillance Division, Ministry of Health, Dakar, Senegal;; ^6^ Department of Medicine, University of Cambridge, Cambridge, United Kingdom;; ^7^ Heidelberg Institute of Global Health, University of Heidelberg, Heidelberg, Germany;; ^8^ Madagascar Institute for Vaccine Research, University of Antananarivo, Antananarivo, Madagascar

## Abstract

Hepatitis E, caused by hepatitis E virus (HEV), is a highly endemic disease with a major public health impact. In Senegal, the first HEV outbreak was identified in 2014 in Kedougou, and a recent study focusing on pregnant women showed a seroprevalence of 7.8%. Apart from these data, very little is known about its burden in Senegal, and there is no specific surveillance program. We describe HEV prevalence among acute febrile jaundice patients in Senegal. A retrospective analysis using jaundice samples (2008–2020) collected through the national yellow fever surveillance program were tested for HEV by IgM/IgG ELISA and reverse transcription polymerase chain reaction (RT-PCR). Sequencing and phylogenetic analyses were performed on RT-PCR-positive samples. Statistical tools were used to analyze the HEV prevalence. In total, 2,387 samples were tested, and 174 IgG, 135 IgM/IgG, and 29 IgM positive samples were detected. Among the IgM-positive samples, 74 were positive by RT-PCR. The results showed an overall HEV prevalence of 14% in febrile jaundice patients in Senegal. Phylogenetic analyses showed the circulation of genotype 2b. Our data confirmed the HEV outbreak in Kedougou in 2014 and showed the detection of acute cases in other regions. We also detected in these regions genotype 2b, which is usually associated with large outbreaks in many African countries, suggesting a high risk of HEV outbreak occurrence in Senegal. Finally, our data showed that the yellow fever surveillance program, which is based on jaundice and already exists in many countries, could be a valuable tool for HEV surveillance.

## INTRODUCTION

Hepatitis E virus (HEV) is the main cause of acute hepatitis in the world, particularly in areas with poor access to safe drinking water and sanitation.^1^^,^^2^ Hepatitis E virus belongs to the Hepeviridae family of the *Paslahepevirus *genus and *Paslahepevirus balayani* species.^3^ Hepatitis E virus is an RNA virus (single stranded and positive sense), and it has eight different genotypes.^4^ Among them, genotypes 1–4 are the main ones causing infections in humans.^5^^,^^6^ Genotypes 3 and 4 are zoonotic, with rare transmission to humans through the consumption of infected meat or drinking water.^7^^,^^8^ They are more prevalent in industrialized and high-income countries.^9^ However, genotypes 1 and 2 mainly circulate in developing countries and infect only humans.^10^ Genotypes 1 and 2 are transmitted by fecal–oral transmission and are often associated with unsanitary conditions and the absence of drinking potable water.^7^ They cause both sporadic infections and large waterborne epidemics.^1^^,^^11^ Genotype 1 and 2 cases are generally asymptomatic, but severe acute forms with jaundice can occur in high-risk populations, such as pregnant women and immunocompromised people, leading to a high mortality rate.^12^^,^^13^

Outbreaks owing to genotypes 1 and 2 are periodically reported in Asia, Africa, Mexico, and the Middle East.^14^^,^^15^ Indeed, large waterborne outbreaks are reported in these regions because of fecal contamination of water supplies, particularly after heavy rainfall and flooding. It has been shown that these genotypes cause about 20 million new infections yearly in Asia and Africa, resulting in 3.4 million symptomatic cases, 70,000 deaths from acute liver failure, and 3,000 stillbirths.^15^^,^^16^

In Africa, between 2010 and 2020, several HEV outbreaks were reported across various countries, with over 30,000 suspected or confirmed cases and more than 610 deaths.^17^^–^^19^ Over 11,000 cases were reported in the largest HEV outbreak, which occurred in Upper Nile, South Sudan between July 2012 and October 2013, with overcrowding, poor sanitation, and flooding being the main risk factors.^19^

For the first time in Senegal, an HEV outbreak was notified in 2014 in the Kedougou region, which is located in the southeast, through investigations of a Rift Valley fever case detected with a febrile syndrome surveillance based in Kedougou. During this epidemic, a particular severity was observed in HEV-positive pregnant women, with a high mortality of 100%.^20^

Recently, a study focused on pregnant women in Senegal has shown an overall seroprevalence for HEV of 7.8%, with higher seroprevalence in Kedougou (where the outbreak occurred) and Saint-Louis, located in northern Senegal.^21^

Hepatitis E virus is, therefore, a significant public health problem in Africa, and a better understanding of its epidemiology will facilitate the implementation of evidence-based control strategies to prevent the spread of the virus. However, HEV is barely covered in Africa, with little data available, and there is no specific control program or surveillance system.

In some African countries, studies have shown that the surveillance of yellow fever (YF) based on febrile jaundice has frequently been the indicator system signaling several HEV cases in African populations.^20^^,^^22^^,^^23^

Indeed, during the last outbreak of febrile jaundice in Darfur, YF surveillance showed the coexistence of YF and HEV infections in affected localities.^22^ In addition, in Burkina Faso and Chad, studies conducted on patients presenting with febrile jaundice have shown global HEV IgG seroprevalences of 18.2% and 34.1%, respectively.^23^^,^^24^

Here, we analyzed the prevalence of hepatitis E in Senegal between 2008 and 2020 using acute jaundice patient samples collected through the national YF surveillance network. Indeed, we conducted serological and molecular testing on the samples and analyze the data by age, sex, year, and region. We also conducted genetic characterization of the circulating HEV strains. We finally discuss the usefulness of YF surveillance in the detection of HEV in Africa, particularly in Senegal.

## MATERIALS AND METHODS

This was a retrospective study carried out on biobanked febrile jaundice samples collected as part of the national YF surveillance program in Senegal from 2008 to 2020.

### Presentation of the YF surveillance program.

National YF surveillance is coordinated by the Ministry of Health with support of the WHO in the context of the African YF laboratory network. Yellow fever surveillance is based in all health districts of the country, and the case definition is any person presenting an acute onset of fever with jaundice appearing within 14 days of the onset of the first symptoms. Thus, in all districts, for any suspected patient, a blood sample is collected, and a case investigation form is filled for shipment to the YF national and regional reference laboratory at Institut Pasteur de Dakar (IPD). The samples are routinely tested at IPD by serology and reverse transcription polymerase chain reaction (RT-PCR) for YF and other viruses that could cause similar symptoms (i.e., dengue, Rift Valley fever, Crimean–Congo hemorrhagic fever, etc.). However, HEV testing is not routinely done in the reference laboratory. The remaining samples are stored at the IPD biobank.

### Samples.

In total, 2,387 samples available in the biobank that were negative for YF and other tested arboviruses and that presented high sample volume (>500 *µ*L) were used for this study regardless of the age, sex, and location of the patients. These samples were anonymized, and the data were obtained only from the YF routine surveillance questionnaire. Therefore, there was no new sampling or additional questions to the YF standardized questionnaire for this study.

### Laboratory testing.

#### Serological testing.

Qualitative IgM (capture) and IgG (indirect) ELISAs were performed on the samples using the Wantai HEV diagnostic kit (v. 2016-01, Beijing Wantai Biological Pharmacy Enterprise Co., Ltd., Beijing, China) following the manufacturer’s instruction.^25^ Briefly, for the IgM ELISA, the microwells were precoated with antibodies directed against human IgM proteins (anti-μ chain) that will capture IgM antibodies in the human sera. After two different washing steps, HEV recombinant ORF2 antigen conjugated to the horseradish peroxidase (HRP) enzyme was added to the wells for revelation with chromogenic substrate (tetramythyl-benzydil [TMB]). For the IgG testing, the microwells were precoated with the HEV ORF2 protein that will bind antibodies present in the human sera. After washing, anti-human IgG antibodies (anti-IgG) conjugated to HRP were then added. Chromogenic solutions containing TMB were finally added. The validity of both tests was analyzed based on criteria recommended by the manufacturer. The test was repeated for any positive sample for confirmation.

#### Molecular testing.

Viral RNA extraction was performed on HEV IgM*-*positive samples by using the QiaAmp Viral RNA Mini Kit according to the manufacturer’s instructions (Qiagen, Hilden, Germany).

The RNA samples were screened for the presence of HEV by RT-PCR using the TaqMan Fast Virus 1-Step Master Mix Kit (Applied Biosystems, Vilnius, Lithuania) and the following primers and probe (Table 1) previously described.^26^^,^^27^ Similarly, we have used primers for human glyceraldehyde 3-phosphate dehydrogenase (GAPDH) (Table 1).^28^ These primers were used as an internal control to confirm that the extracted samples contained sufficient levels of RNA and had not been degraded during storage. Thus, all samples must be positive for human GAPDH to be considered for HEV analyses.

**Table 1:** Primers and probes for hepatitis E virus and human glyceraldehyde 3-phosphate dehydrogenase

Target	Primer/Probe Name	Primer/Probe Sequence
HEV	HEV_F	GGTGGTTTCTGGGGTGAC
	HEV_R	AGGGGTTGGTTGGATGAA
	HEV_probe	FAM-TGATTCTCAGCCCTTCGC-BHQ1
Human GAPDH	GAPDH_F	CCACCCATGGCAAATTCC
	GAPDH_R	TCGCTCCTGGAAGATGGTG
	GAPDH_probe	TAMRA-TGGCACCGTCAAGGCTGAGAACGT-BHQ2

GAPDH = glyceraldehyde 3-phosphate dehydrogenase; GAPDH_F = glyceraldehyde 3-phosphate dehydrogenase forward (forward primer); GAPDH_R = glyceraldehyde 3-phosphate dehydrogenase reverse (reverse primer); HEV = hepatitis E virus; HEV_F = hepatitis E virus forward (forward primer); HEV_R = hepatitis E virus reverse (reverse primer).

Six HEV-positive samples with threshold cycle values less than or equal to 28 were selected for sequencing using the hybridization-based capture method with the Twist Comprehensive Panel Kit (103550_Twist) as recommended by the manufacturer (Twist Bioscience HQ, South San Francisco, CA). Libraries were sequenced on an Illumina Miseq platform (Illumina, San Diego, CA).

The sequencing reads were analyzed using the EDGE bioinformatics pipeline (https://www.edgebioinformatics.org/; accessed January 5, 2022), which generated a single Fasta file for each sample. The generated genomes during this work were submitted to public database BLAST to identify their homologous sequences and then, combined with a representative subset of 24 HEV sequences previously available in GenBank. Sequences were cleaned using BioEdit software (v. 7.0.5.3) and aligned using the Mafft program.^29^ The maximum likelihood (ML) phylogenetic tree was constructed using the IQ-Tree software^30^ with the best-fitted model to our dataset. The ML tree was visualized using the iTOL software.^31^

## STATISTICAL ANALYSES

All of the data obtained from the anonymized patients as well as the laboratory results were compiled in an Excel spreadsheet v. 2019 (Microsoft Corp, Redmond, WA) and then analyzed with the R software under R Studio v. 1.3.959 (R Foundation, Vienna, Austria). Demographic factors (age and sex) associated with HEV exposure were assessed using bivariate analyses. Indeed, our population was stratified by 5 years based on a previous study.^32^ The Fisher test was used with a significance threshold *P*-value of <0.05.

## RESULTS

### Sociodemographic characteristics of the study population.

We analyzed a total of 2,387 sera samples collected from all 14 regions of Senegal through the YF surveillance system from 2008 to 2020. The highest number of samples (357) was collected in 2014 (Table 2). The male to female sex ratio was 1.5 (1,389 males and 936 females). The average age of the population was 19 years old, with an SD of 15.98, and the median age was 15 years old, ranging from 0 to 92 years old. The most prevalent age group was 15–25 years old, constituting 20% of the study population. The Dakar region had the highest representation with 376 samples followed by the Kedougou region with 271 samples (Table 3).

**Table 2:** Number of samples collected by year

Years	2008	2009	2010	2011	2012	2013	2014	2015	2016	2017	2018	2019	2020
Numbers (%)	80 (3.4)	188 (7.9)	38 (1.6)	43 (1.8)	113 (4.7)	149 (6.2)	357 (15.0)	199 (8.3)	236 (9.9)	249 (10.4)	232 (9.7)	253 (10.6)	250 (10.5)

**Table 3:** Sociodemographic characteristics of the study population

Characteristics	Numbers
Sex, %	
Male	1,389 (60)
Female	926 (40)
Missing	72
Age, years	
Mean (SD)	19 (15.9)
Median (minimum to maximum)	15 (0–92)
Missing	159
Age group, years, %	
<5	327 (14.7)
5 to <10	438 (19.7)
10 to <15	346 (15.5)
15 to <20	283 (12.7)
20 to <25	203 (9.1)
25 to <30	151 (6.8)
30 to <35	124 (5.6)
35 to <40	86 (3.9)
40 to <45	54 (2.4)
45 to <50	54 (2.4)
≥50	162 (7.3)
Missing	159

### Detection of HEV in the study population.

**Figure 1.**
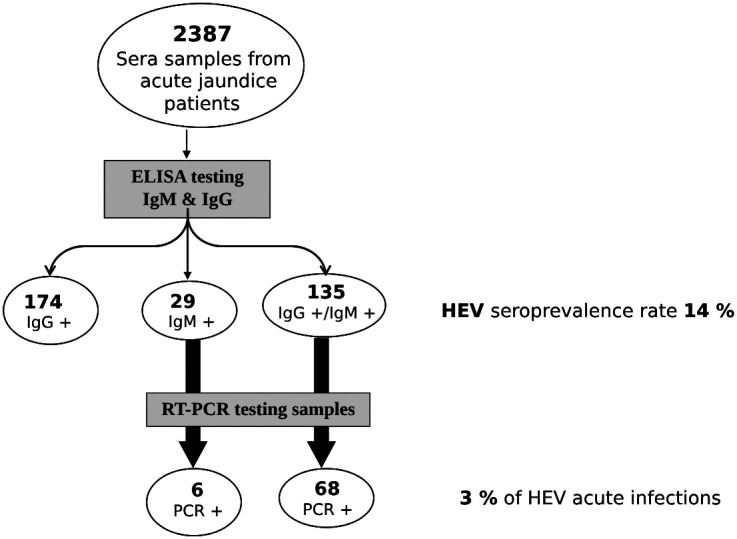
Algorithm test and detection of the hepatitis E virus (HEV) by reverse transcription polymerase chain reaction (RT-PCR) and ELISA in the study population. PCR = polymerase chain reaction.

Out of the 2,387 sera samples tested, 174 IgG, 135 IgM/IgG, and 29 IgM HEV-positive sera were detected by ELISA. In addition, among the IgM-positive samples (164, including 135 positive for both IgM and IgG and 29 positive for IgM only), 74 RT-PCR HEV-positive samples were detected, showing acute infections (Figure 1). These results showed an overall HEV prevalence of 14% among the acute jaundice patients in Senegal between 2008 and 2020.

Table 4 shows that males had overall higher exposure rates compared with females, with 197 (8.5%) for IgG and 114 (4.9%) for IgM in males versus 104 (4.4%) for IgG and 45 (1.9%) for IgM in females, respectively. This trend also extended to HEV-positive cases detected by RT-PCR, with 47 (1.9%) cases in males compared with 25 (1.0%) cases in females. However, the differences were not statistically significant (*P* = 0.1907), except for IgM (*P* = 0.01096).

**Table 4:** Prevalence of hepatitis E by gender

Characteristics	*N*	Anti-HEV IgG+, *n* (%)	*P*-Value	Anti-HEV IgM+, *n* (%)	*P*-Value	HEV RT-PCR+, *n *(%)	*P*-Value
Male	1,389 (58.2)	197 (8.5)	0.1907	114 (4.9)	0.01096	47 (1.9)	0.2011
Female	926 (38.8)	104 (4.4)		45 (1.9)		25 (1.04)	
Undetermined	72 (3.0)	8 (0.3)		5 (0.2)		2 (0.08)	
Total	2,387 (100)	309 (13.0)	–	164 (7.0)	–	74 (3.1)	–

HEV = hepatitis E virus; *N* = total sample size; *n* = number of positive samples; RT-PCR = reverse transcription polymerase chain reaction.

**Figure 2.**
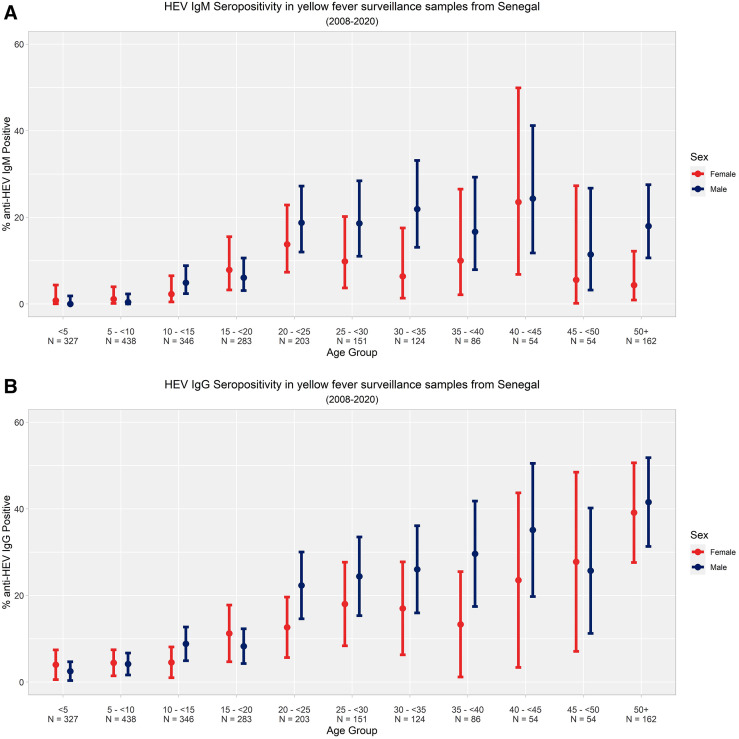
Age- and sex-stratified (**A**) IgM and (**B**) IgG seropositivity. On the *y* axes, we have the percentages of (**A**) antihepatitis E virus (anti-HEV) IgM and (**B**) anti-HEV IgG antibodies. On the *x* axes, we have the age ranges in 5-year groups from younger than 5 years old to 50 years old and older. The red and blue bars represent the percentages of anti-HEV IgG/IgM antibodies for women and men, respectively, across the age groups.

Hepatitis E virus IgG seroprevalence stratified by sex and age group showed clear increasing rates for both males and females according to age, with patients between 25 and 50 years old being the most affected (Figure 2A). However, for HEV IgM seroprevalence (Figure 2B), corresponding to recent infections, the data showed variations between age groups in both males and females. In addition, males showed the highest rates in patients ages between 20 and 45 years old and older than 50 years old, whereas females showed the highest rates in patients ages between 20 and 25 years old and between 40 and 45 years old. For both IgG and IgM, the data showed low seroprevalence in children and young adults younger than 20 years old. Likewise, the differences in positivity between the age groups are significantly different, with *P*-values of <2.2e^−16^ for both IgG/IgM.

### Temporal distribution of hepatitis E cases.

**Figure 3.**
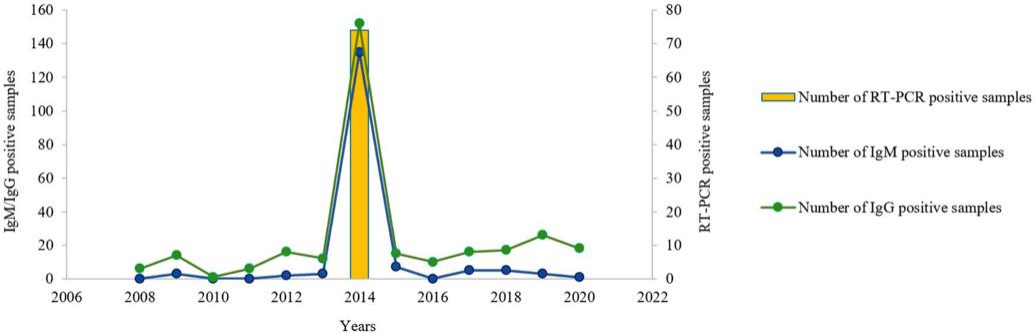
Distribution of hepatitis E virus (HEV) IgM/IgG-positive and reverse transcription polymerase chain reaction (RT-PCR)-positive cases over time. The left *y* axis represents the number of samples positive for anti-HEV IgM/IgG. The right *y* axis represents the number of samples positive by RT-PCR. The *x* axis shows the years ranging from 2006 to 2022. The blue and green curves represent the numbers of IgM- and IgG-positive samples, respectively, over the years. The yellow bar indicates the number of RT-PCR-positive samples by year.

During the study period, the highest numbers of new cases detected both with IgM (135 positive cases, 82.3%) and with RT-PCR (74 positive cases, 100%) were observed in the year 2014. Similarly, the number of IgG-positive cases (*n* = 152, 49%) indicating previous infections was also higher in 2014 (Figure 3); IgG-positive cases were also observed in 2009, 2012, and during the period between 2016 and 2020. Reverse transcription polymerase chain reaction-positive cases were only detected during the year 2014 during our study.

### Spatial distribution of hepatitis E cases in Senegal.

**Figure 4.**
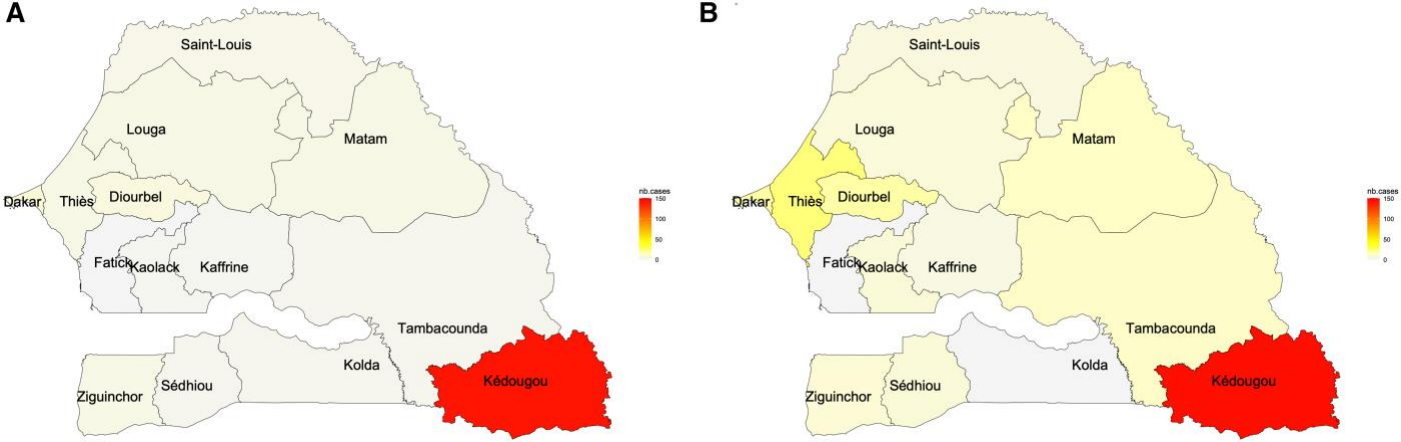
Distribution of hepatitis E virus (HEV) (**A**) IgM- and (**B**) IgG-positive cases according to the regions in Senegal. This figure shows the number of samples positive for anti-HEV IgM and IgG based on a scale from 0 to 150 across all regions of Senegal. For HEV IgM-positive cases, nb = 164; for HEV IgG-positive cases, nb = 309. nb = total number of positive samples.

Our data showed that HEV antibodies were detected in 12 of the 14 regions in Senegal, and the Kedougou region exhibited the highest prevalence, with 137 IgM-positive cases (83.5%), 153 IgG-positive cases (49.5%), and 73 RT-PCR-positive cases (98.6%). Following closely are the regions of Dakar (6 IgM positive and 20 IgG positive between 2008 and 2020), Diourbel (6 IgM positive, 20 IgG positive, and 1 RT-PCR positive between 2008 and 2020), Thies (2 IgM positive and 26 IgG positive between 2008 and 2020), Matam (3 IgM positive and 14 IgG positive between 2008 and 2020), and Tambacounda (2 IgM positive and 12 IgG positive between 2009 and 2020) (Figure 4).

### Genetic characterization of HEV viral genome: Phylogenetic analyses.

Of the 74 RT-PCR-positive samples, only 6 underwent sequencing, resulting in the generation of three complete genome sequences, including one from Diourbel (SEN_DIO_2014) and two from Kedougou (SEN_KED_2014 and SEN_KED2_2014). These newly characterized HEV sequences from Senegal were deposited in GenBank under accession numbers PP271982, PP271983, and PP271984, respectively.

**Figure 5.**
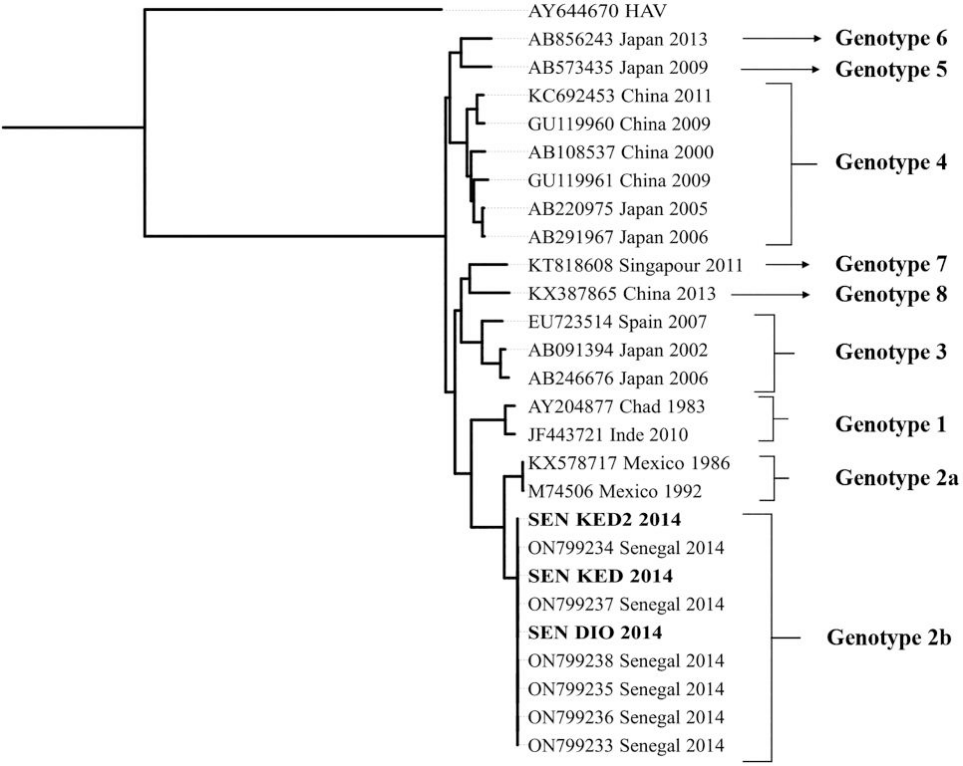
Maximum likelihood tree of hepatitis E virus strains based on the complete genome. Our strains SEN_DIO_2014 (accession number PP271982), SEN_KED_2014 (accession number PP271983), and SEN_KED2_2014 (accession number PP271984) are indicated with boldface.

The ML tree was constructed using the IQ-Tree software^30^ for 1,000 bootstrap replications with the TIM2+F+R8 model and visualized using the iTOL software.^31^ The three newly characterized HEV isolates from Senegal were closely related and grouped with previous sequences in the Kedougou region during the 2014 outbreak.^20^ The phylogenetic tree confirmed their classification within HEV genotype 2b (Figure 5).

## DISCUSSION

In this study, we used samples from the national YF surveillance system based on jaundice to assess the prevalence of hepatitis E in Senegal. Analysis of samples collected between 2008 and 2020 revealed the circulation of genotype 2b and an overall prevalence of 14%. A similar study conducted in Burkina Faso between 2013 and 2016 focusing on patients with febrile jaundice reported a prevalence of 18.2%, slightly higher than that found in our study.^23^ This could be attributed to the longer study period (2008–2020) and the larger number of samples tested in our study, indicating significant exposure of West African populations to HEV. Moreover, in central Africa, particularly in Chad, a study also based on patients with febrile jaundice enrolled through the YF surveillance program has showed an overall seroprevalence of 34.1%.^24^ This also showed high exposure of central African populations to HEV.

Apart from seroprevalence studies on acute jaundice patients, other seroprevalence studies have also been conducted in Africa but were focused on specific groups, such as pregnant women, hemophiliacs, hemodialysis patients, or blood donors. Indeed, a seroprevalence study that focused on pregnant women in Senegal in 2021 showed overall lower seroprevalence (7.8%).^21^ In our study, the inclusion of acute jaundice samples collected in 2014 when the HEV 2014 epidemic occurred in Kedougou probably led to an overestimation of the seroprevalence, particularly in this region. However, similarly to our study, the Kedougou region showed in the seroprevalence study with pregnant women the highest seroprevalence and the detection of acute HEV IgM-positive cases, confirming the active and high circulation of HEV in this area, even after the 2014 epidemic.^21^ Indeed, the rapid proliferation of traditional gold mining sites in the Kedougou region has led to massive migration of people from neighboring West African countries and the establishment of several small villages where poor hygiene and sanitation conditions exist, and this is probably contributing to this high HEV circulation.

Our study also detected for the first time unnoticed cases of acute HEV (IgM positive/negative and RT-PCR) in other regions (Dakar, Thies, and Diourbel). Those cases were detected in 2014 during the HEV outbreak in the Kedougou region and could probably be imported cases as jaundice clusters or deaths in pregnant women were not notified in these regions in 2014. However, the study conducted in Senegal has also shown exposure of pregnant women to HEV in Dakar as well as in two other regions: Saint-Louis (in the north) and Ziguinchor (in the southwest).^21^ This confirms HEV circulation in Dakar and other regions in Senegal, and it emphasizes the need to reinforce surveillance for timely detection of the HEV cases and the implementation of efficient responses to avoid outbreaks.

Our study showed a higher prevalence of HEV in men, consistent with findings in Burkina Faso and the Central African Republic (CAR).^23^^,^^33^ Indeed, in 2010, Goumba et al.^33^ reported a higher level of anti-HEV IgG (85.1%) in men compared with women (73.8%) in CAR. This could be attributed to the mobility of men working in high-risk areas, making them more susceptible.^4^

Our data also demonstrated that individuals between 15 and 50 years old are the most affected ones. Similar data were also obtained in Burkina Faso, aligning with the prediction of a higher number of symptomatic genotypes 1 and 2 infections in young adults compared with children in developing countries.^23^^,^^34^ The mechanism explaining this age-related difference in symptomatic cases with regard to HEV genotype remains unknown and warrants further investigations.

Furthermore, our study identified the circulation of genotype 2b in Kedougou, consistent with recent data from Kedougou’s epidemic under the febrile syndrome surveillance system.^20^ Genotype 2b was also detected for the first time in the Diourbel region in our study using febrile jaundice monitoring. This indicates that the majority of acute infections in Senegal are attributed to this genotype, suggesting a fecal–oral mode of transmission. Genotype 2b has also been reported in several countries recently, including Burkina Faso,^23^ suggesting the risk of occurrence of major epidemics in Africa and the necessity to enhance surveillance in high-risk areas in Africa. Nevertheless, all RT-PCR-positive samples were collected in 2014, which represents a major limitation of our study regarding the representativeness of the strains that circulated in Senegal.

Here, the use of YF surveillance allowed us to confirm the HEV outbreak in Kedougou and detect sporadic cases in other regions. Interestingly, our methodology showed the importance of combining ELISA and RT-PCR testing for the detection of HEV cases. Indeed, as the virus lasts longer in the stool than in the blood, using IgM ELISA on a blood sample as the primary test is more efficient than RT-PCR for HEV detection.

Similarly to our study, febrile jaundice surveillance was used in Burkina Faso and Chad, leading to the detection of HEV cases between 2013 and 2019.^23^^,^^24^ This suggests that the existing YF surveillance system that is already in place in several African countries could serve as a valuable tool for detecting HEV cases in Africa. Indeed, instead of establishing a new HEV-specific surveillance, which could be costly for countries and their partners, such as the WHO, including IgM ELISA and RT-PCR tests for HEV into the differential diagnostics of YF could provide a rapid and efficient tool for identifying HEV cases and implementing appropriate response strategy. This approach would also contribute to a better understanding of HEV’s epidemiology in Africa and facilitate the development of effective control strategies.

## CONCLUSION

The study of the prevalence of hepatitis E from 2008 to 2020 confirms the circulation of HEV and proves that it can be an important cause of acute febrile jaundice in Senegal. Hence, hepatitis E warrants more consideration in Senegal, particularly for patient with unexplained hepatitis, especially in the eastern, western, and central regions. Utilizing the YF surveillance system in Senegal, we were able to show virus circulation in different regions and confirm the presence of genotype 2b, suggesting a potential risk of major epidemics in Senegal because of the virulence of this genotype, which underscores the necessity for enhancing the surveillance system. Finally, our findings suggest the effectiveness of HEV detection through the existing YF surveillance, implying that this system that is present in several African countries, including Senegal, could be serve a valuable tool for HEV monitoring.
